# Prevalence and zoonotic potential of canine hookworms in Malaysia

**DOI:** 10.1186/1756-3305-5-88

**Published:** 2012-05-07

**Authors:** Mohammed AK Mahdy, Yvonne AL Lim, Romano Ngui, MR Siti Fatimah, Seow H Choy, Nan J Yap, Hesham M Al-Mekhlafi, Jamaiah Ibrahim, Johari Surin

**Affiliations:** 1 Department of Parasitology, Faculty of Medicine, University of Malaya, 50603 Kuala Lumpur, Malaysia; 2 Department of Parasitology, Faculty of Medicine, Sana’a University, Sana’a, Yemen

**Keywords:** Hookworm, Dogs, *A. ceylanicum*, *A. caninum*, Malaysia

## Abstract

**Background:**

Canine hookworm infection is endemic in Southeast Asian countries with a prevalence ranging from 70% to 100%, with zoonotic transmission representing a potentially significant public health concern. However, there are limited data available on the prevalence of canine hookworms in Malaysia. This study was conducted to determine the prevalence of hookworm and *Ancylostoma* species among dogs in Malaysia.

**Methods:**

Faecal samples were collected from 221 dogs living in urban areas, rural areas and animal shelters in Selangor. Faecal samples were processed using the formal-ether concentration technique followed by wet mount preparation and iodine staining for the detection of hookworm eggs. Samples positive for hookworm eggs were examined using PCR, targeting ITS2 and 28 s rRNA region, and subsequently sequenced in both directions. The sequences were phylogenetically analysed using MrBayes for Bayesian Inference.

**Results:**

The overall prevalence of hookworm among dogs was 48% (95%CI; 41.41–54.95). Rural stray dogs had the highest prevalence 71.4% (95%CI; 61.13–81.49) followed by urban stray dogs, recording 48% (95%CI; 34.15–61.85) and lastly dogs in shelters with 28.7% (95%CI; 19.56–37.84). Logistic regression identified rural stray dogs as a high risk group (OR = 4.55, 95%; 2.50–8.31) and keeping dogs in shelters as a protective factor (OR = 0.24, 95%; 0.14–0.43). Molecular methods identified both *Ancylostoma ceylanicum* and *Ancylostoma caninum* with *A. ceylanicum* being predominant among urban stray dogs. Rural dogs had a higher prevalence of *A. caninum* than *A. ceylanicum,* while both species showed equal distribution among dogs in shelters. Phylogenetic analysis placed *A. ceylanicum* isolated from dogs in one group with *A. ceylanicum* human isolates.

**Conclusion:**

This study indicates that dogs have the potential to act as reservoir hosts of human hookworm infection in Malaysia. This finding necessitates the inclusion of dogs in any interventions to combat hookworm in the country.

## Background

Canine hookworm infection is endemic in Southeast Asian countries with a prevalence ranging from 70% to 100%, with zoonotic transmission representing a potentially significant public health concern [1-3]. Dog hookworms include *Ancylostoma caninumAncylostoma brazilienseAncylostoma ceylanicum* and *Uncinaria stenocephala*. Of these species, only *A. ceylanicum* develops to adult in humans, causing patent enteric hookworm infection [4,5]. On the other hand, *A. caninum*, occasionally reaches adulthood in humans, causing eosinophilic enteritis [6-8].

Although *A. braziliense* is the predominant species which causes hookworm related cutaneous larva migrans (CLM), the late larval stage of *A. caninum* has been implicated in CLM [9], leading to the lesser common follicular dermatitis [10-12]. Caninum larval stage has also been suspected as a cause of diffuse unilateral subacute neuroretinitis [13] and has been found intracellularly within muscle fibers [14]. Hookworm-related CLM cases have been reported from Malaysian patients [15-19] and in tourists who have visited Malaysia [20-22].

Although human hookworm infections in Malaysia continue to cause significant morbidity, particularly among disadvantaged communities [23-28], there are limited data available on the prevalence of canine hookworms [29-31]. A recent study among human populations using molecular tools showed that *A. ceylanicum* represented 23.4% of human hookworm infections. The study not only isolated *A. ceylanicum* from humans but also from dogs living in close contact with these humans and statistical analysis identified contact with dogs and cats as a significant predictor of human hookworm infections [32]. The present study will be applying molecular tools in combination with classical parasitological methods to determine the prevalence and genetically characterize canine hookworm from different locations (i.e., urban areas, rural areas and animal shelters). This study will unfold the potential role of dogs as possible reservoir hosts of human hookworm infections.

## Methods

### Samples and microscopy

**Figure 1:**
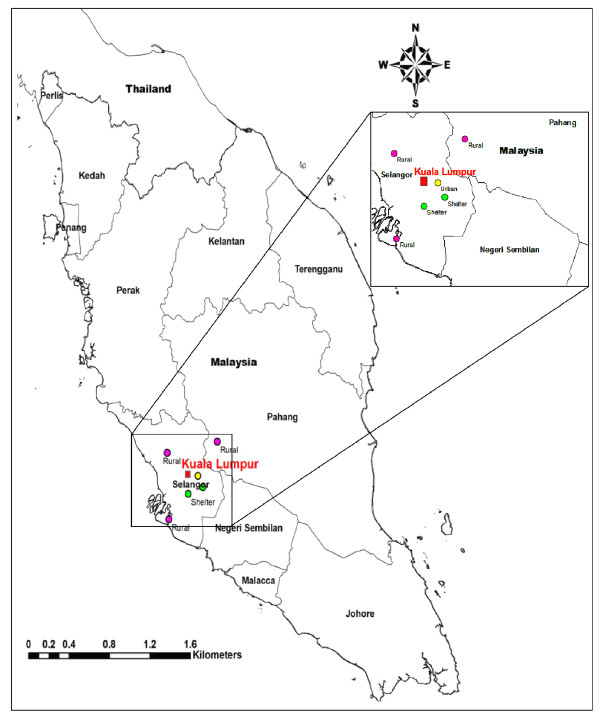
Map of the sampling areas.

The study was conducted among dogs from three different locations, which included rural stray dogs in Selangor and Pahang, urban stray dogs from Kuala Lumpur and dogs from animal shelters in Selangor (Figure 1). Compared to the stray dogs in rural and urban communities, dogs in animal shelters do undergo periodic deworming.

Faecal samples were collected in wide mouth and screw capped faecal containers with attached scoops, which were properly labelled. The collected faecal samples were transported to the Department of Parasitology, Faculty of Medicine, University of Malaya for further analysis in the same day. Stool samples were processed using the formalin-ether concentration technique. Direct wet mount was prepared by mixing a small amount (approximately 1 gram) of faeces in a drop of iodine on a clean and dry slide. The smear was covered with a cover slip and examined under a light microscope starting from low power (10x) to high power magnification (40x) for the presence of hookworm-like eggs.

### Molecular analysis

Genomic DNA was extracted directly from positive faecal sample using PowerSoil DNA Kit (Mo Bio, cat. no. 12888–100, CA, USA) according to manufacturer’s instructions. Final elution of DNA was made in 50 μl of elution buffer instead of 200 μl as recommended by the manufacturer. The extracted DNA was stored at −20°C until required for PCR amplification.

A partial region (≈ 310 bp) of internal transcribed spacer ITS-2 and 28 S rRNA region of *Ancylostoma* spp. was amplified using a forward primer NC1 (5′-ACG TCT GGT TCA GGG TTC TT-3′) and reverse primer NC2 (5′-TTA GTT TCT TTT CCT CCG CT-3′). Control samples without DNA (negative control/DNA blank) and with hookworm genomic DNA (positive control) were included in each PCR run. The PCR was carried out in a 50 μl final PCR mixture containing 1X PCR buffer, 200 μM dNTPs, 1 mM MgCl_2,_ 10 pmol of each primer, 5 U *Taq* polymerase and 6 μl of DNA template. The cycling conditions were as follows: denaturation at 94°C for 5 min, followed by 30 cycles of 94°C for 30 s (denaturing), 55°C for 30 s (annealing), 72°C for 30 s (extension) and a final extension at 72°C for 7 min. PCR products were subjected for electrophoresis on 1.2% agarose gel and stained with Cybersafe stain.

**Figure 2:**
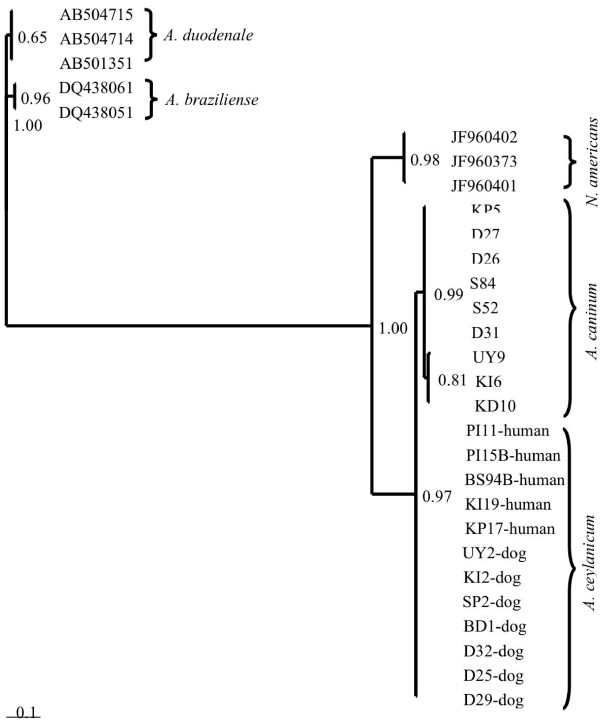
**A phylogenetic tree based on partial ITS2 sequences of hookworm species constructed using Mrbayes program.** Figures on the branches are bootstrap probabilities (in %) based on 1000 bootstrapped trees. Accession numbers indicate sequences from the GeneBank database. Sequences from *A. ceylanicum* infecting humans were received from a previous study in Malaysia (Ngui *et al*., 2012).

The amplicons were purified using QIAquick Gel Extraction Kit (QIAgen, cat. no. 28104, Hilden, Germany) according to the manufacturer’s instructions. All the purified amplicons were sent for DNA sequencing in both directions using forward and reverse primers with an ABI 3730XL sequencer (Bioneer Corporation, South Korea). Sequence chromatograms were viewed using Sequence Scanner version 1.0 programme (Applied Biosystems, USA). Forward and reverse sequences were edited, manually aligned and the consensus sequence was created for each sample using the BioEdit Sequence Alignment Editor Programme (http://www.mbio.ncsu.edu). The similarity between each consensus sequence was then compared to previously published sequences of hookworm using the Basic Local Alignment Search Tool (BLAST) (http://blast.ncbi.nlm.nih.gov). Sequences generated in this study were deposited in GenBank, under the accession numbers (JQ673419 - JQ673426). Phylogenetic analysis was carried out by Bayesian Inference using Monte Carlo Markov Chain (MCMC) in MrBayes 3.1.2 [33]. The General Time Reversible (GTR) model of evolution was used for nucleotide substitution [34] with gamma distribution rate. Reference sequences from previous published studies were selected for phylogenetic analysis (Figure 2). The percentage of replicate trees in which the associated hookworm isolates were clustered together in the bootstrap test values (1000 replicates) is shown next to the branches.

### Statistical analysis

Data were analyzed using SPSS programmed for windows version 11.5 (SPSS Inc., Chicago.IL, USA). Univariate and multivariate analysis using logistic regression were applied to identify risk factors. Chi-square was used to investigate the association between variables. The significance was defined as *p* < 0.05.

## Results

### Prevalence and distribution

A total of 221 faecal samples were collected from dogs consisting of 94 samples from dog shelters, 77 samples from rural stray dogs [32] and 50 from urban stray dogs. The overall prevalence of hookworm through microscopy detection was 48% (95%CI; 41.41–54.95). The prevalence was the highest among rural stray dogs (71.4%, 95%CI; 61.13–81.49) followed by urban stray dogs (48%, 95%CI; 34.15–61.85) and finally dogs in shelters (28.7%, 95%CI; 19.56–37.84) (Table1). Multivariate analysis showed that rural stray dogs are at a five fold higher risk of hookworm infection compared to dogs living in other locations (OR = 4.55, 95%; 2.50–8.31). Keeping dogs in shelters appeared to be a protective factor (OR = 0.24, 95%; 0.14–0.43) (Table2).

**Table 1:** Prevalence of hookworm infection among dogs according to locations

Variable	Examined n	Infected n (%)	95% CI
Rural stray dogs	77	55 (71.4)	61.31 – 81.49
Urban stray dogs	50	24 (48)	34.15 – 61.85
Dogs in shelters	94	27 (28.7)	19.56 – 37.84
Total	221	106 (48)	41.41 – 54.95

**Table 2:** Univariate and multivariate analysis of the association of hookworm with locations (n = 221)

Variable		Examined (n)	Infected n (%)	OR (95% CI)
Rural stray dogs*	Yes	77	55 (71.4)	4.55 (2.50 – 8.31)
	No	144	51 (35.4)	1
Urban stray dogs	Yes	50	24 (48.0)	1.00 (0.53 – 1.88)
	No	171	82 (48.0)	1
Dogs in shelters*	Yes	94	27 (28.7)	0.24 (0.14 – 0.43)
	No	127	79 (62.2)	1

* Confirmed by stepwise conditional logistic regression.

### Species identification and distribution

Specimens positive for hookworm using microscopy (106) were subjected to PCR followed by DNA sequencing based on a partial region (≈ 310 bp) of ITS2 and 28 s rRNA for species identification. Of these, 82 were PCR positive and successfully sequenced. Comparison of these sequences with hookworm sequences deposited in the GenBank database using BLAST showed that 52% (43 of 82) were *A. ceylanicum* and 48% (39 of 82) were *A. caninum.* The study indicted that *A. ceylanicum* was predominant among urban stray dogs and equally distributed with *A. caninum* in dogs living in shelters. In rural communities, stray dogs were infected more with *A. caninum* than *A. ceylanicum* (Table3).

**Table 3:** **The distribution of** ***Ancylostoma*** **species among dogs based on locations**

		Prevalence n (%)			
	Examined	*A. ceylanicum*	*A. caninum*	*χ*^2^	P value
Dogs in shelters	16	8 (50.0)	8 (50.0)	0.047	P > 0.05
Urban stray dogs	21	16 (76.2)	5 (23.8)	6.38	P < 0.05
Rural stray dogs	45	19 (42.2)	26 (57.8)	4.17	P < 0.05
Total	82	43 (52.4)	39 (47.6)		

### Phylogenetic analysis

Sixteen sequences from this study representing the three groups of dogs (i.e., dogs in shelters, urban stray and rural stray dogs) together with 8 reference sequences obtained from the GenBank database and 5 sequences representing *A. ceylanicum* isolated from humans in Malaysia [32] were used in the phylogenetic analysis using MrBayes software for Bayesian Inference (Figure 2). *A. ceylanicum* sequences isolated from humans and dogs were grouped together with strong support (0.97). *A. caninum* sequences representing the three groups of dogs were placed in one cluster with 0.99 bootstrap support. Sequences representing *A. duodenale, A. braziliense* and *N. americanus* were grouped in separated clusters.

## Discussion

The overall prevalence of hookworm infections among dogs based on single faecal sampling was 48% in this study with rural stray dogs having the highest infection rate, followed by urban stray dogs and finally dogs in shelters. These findings are similar to another previous study carried out among dogs in rural and urban areas in Malaysia in which the prevalence of hookworm infection was 45%. However, in the previous study by Azian *et al*. [29], it was found that urban dogs had the highest infection rate of hookworm. This statement was further assured by the higher contamination of soil from urban areas in the same study. This is most likely due to the presence of stray dogs which scavenge rubbish and defecate all over the places [29].

In Thailand, the prevalence of hookworm infection based on microscopy was 58.9% among dogs in temple communities [2]. In China, 66.3% of dogs in 175 farms in Heilongjiang Province had hookworm infections [35]. In rural areas in India, the prevalence of hookworm ranges from 93% to 98% [36,37]. The high hookworm-infected stray dogs that wander and defecate in the public areas increase the risk of contamination of streets, public parks, playground and beaches with hookworm larvae. The risk of the environmental contamination was confirmed by the detection of hookworm larvae of animal origin in sand samples from Caribbean beaches [38]. It was also found that all travelers who visited Northeast Brazil had acquired hookworm-related CLM during their stay at the beaches [39]. Furthermore, soil contamination with hookworm larvae caused outbreaks of CLM in kindergartens [40], public parks [41], schools [42] and among children playing with sand boxes [43].

Multivariate analysis indicated that rural stray dogs are more exposed to hookworm infection and keeping them in shelters protects them from the infection. High rates among rural dogs could be attributed to contaminated soil in the rural areas with hookworm larvae of free-roaming dogs and cats. Soil contamination could also be aggravated by poor human sanitation behavior. In addition, the soil in most rural communities is muddy and moist which favours the development of hookworm larvae [44]. The present study identified that keeping dogs in shelters reduces that chance of dogs acquiring hookworm infection, as they will be less likely to be exposed to the infection compared to stray dogs and will undergo regular de-worming.

In the present study, both *A. ceylanicum* and *A. caninum* have been isolated from dogs. Phylogenetic analysis using sequences of *A. ceylanicum* isolated from dogs and reference sequences of *A. ceylanicum* isolated from humans placed all dogs and human isolates in one group with high bootstrap support (Figure 2). This clustering indicates that *A. ceylanicum* may play a part in the transmission of human hookworm in Malaysia. Due to the inability to differentiate between species causing human hookworm based on egg morphology, most of the previous studies carried out in Malaysia did not identify the species [23-27]. Thus, it is likely that *A. ceylanicum* has been overlooked in the aetiology of human hookworm in Malaysia.

However, a recent study based on molecular methods, conducted in the rural community reported *A. ceylanicum* as the second species causing human hookworm infection [32]. The ability of *A. ceylanicum* to produce patent infections in humans has been reported [2,3,5,45,46]. In addition, molecular study conducted in Laos showed that one third of human hookworm infections are caused by *A. ceylanicum* and this species was responsible for 85% of hookworm infections among dogs [3]. The detection of *A. ceylanicum* in humans and dogs was also reported in a recent study conducted in Laos [47]. In a survey conducted in Thailand, *A. ceylanicum* was recorded as the predominant species causing hookworm in dogs and was detected in humans. In the same study, only human cases infected with *A. ceylanicum* suffered chronic abdominal disturbance [2]. These reports of *A. ceylanicum* infecting humans and dogs in the same community highlight the important role of dogs in the natural transmission of human hookworm in Southeast Asian countries which warrant further investigations. Therefore, the role of dogs in human hookworm transmission should be considered during the implementation of intervention measures to combat the disease, such as, mass chemotherapy which should be combined with a proper dog management programme for effective control.

In the present study, *A. caninum* was found in 48% of the infected dogs. This finding is concurrent with reports from Thailand [2], India [36] and Australia [48]. *Ancylostoma caninum* can establish pathogenic human infections dependent on the migration of L3 to the ectopic site in the human host. Of them, CLM is the most common clinical manifestation [9]. There are limited reports of CLM among local populations in Malaysia [15-19]. Yap found that of 31 patients with CLM referred to Kuala Lumpur general hospital, only 45.2% had the correct diagnosis by referred primary care physicians [15]. Missing the diagnosis of CLM for other pruritic skin manifestation might underestimate the actual incidence of CLM. Among travelers returning from Malaysia, CLM was diagnosed in 13 cases of 42 patients with dermatologic diagnosis in the period from 1997 to 2006 [20]. However, it is not clear which *Ancylostoma* species caused these CLM cases. Hookworm-related CLM is usually caused by *A. brazilienseA. caninumA. tubaeforme* and *Uncinaria stenocephala*[9,49]. Since no reports of *A. tubaeforme* and *U. stenocephala* in Malaysia and only one case of *A. braziliense* was reported to date [32], we assume that *A. caninum* might be the potential species which caused the hookworm-related CLM in this country.

## Conclusion

This study showed high prevalence of hookworm among dogs in Malaysia especially among stray dogs. This highlights the risk of these infected dogs contaminating public areas with hookworm larvae. The identification of *A. ceylanicum* indicates that dogs may represent reservoir hosts of human hookworm since this species has been confirmed to cause patent infection in humans which warrant further studies on human and dogs in the same location. The high prevalence of *A. caninum* shows that people are at risk of getting hookworm-related CLM, which should be considered in the diagnosis of pruritic skin manifestations. Dogs should be considered as a significant source of human hookworm infection in Malaysia and included in the parasite control programme to minimize the shedding of hookworm eggs. It is crucial that preventive measures such as preventing animals from defecating in the public areas and beaches, cleaning up animal waste and educating people to wear protective footwear while they are in contact with soil or sandy beaches be strictly implemented especially in public places.

## Competing interests

The authors declare that they have no competing interests.

## Authors’ contributions

MAKM, YALL and JS designed the study; MRSF, SHC, RN and NJY did the field work and the lab work; MAKM and HMA analyzed the data; MAKM drafted the manuscript; MAKM, YALL, RN, HMA and JS revised the manuscript. All authors have read and agreed with the content of the submitted manuscript.
